# Engineering a vascularised 3D *in vitro* model of cancer progression

**DOI:** 10.1038/srep44045

**Published:** 2017-03-09

**Authors:** Tarig Magdeldin, Víctor López-Dávila, Judith Pape, Grant W. W. Cameron, Mark Emberton, Marilena Loizidou, Umber Cheema

**Affiliations:** 1 UCL Institute of Orthopaedics and Musculoskeletal Sciences, UCL Division of Surgery and Interventional Science, Stanmore Campus, HA7 4LP, UK; 2 UCL Division of Surgery and Interventional Science, Royal Free Campus, London, NW3 2QG, UK; 3 Sartorius Stedim Biotech, Royston, Herts, SG8 5WY, UK

## Abstract

The hallmark of tumours is the ability of cancerous cells to promote vascular growth, to disseminate and invade to distant organs. The metastatic process is heavily influenced by the extracellular matrix (ECM) density and composition of the surrounding tumour microenvironment. These microenvironmental cues, which include hypoxia, also regulate the angiogenic processes within a tumour, facilitating the spread of cancer cells. We engineered compartmentalized biomimetic colorectal tumouroids with stromal surrounds that comprised a range of ECM densities, composition and stromal cell populations. Recapitulating tissue ECM composition and stromal cell composition enhanced cancer cell invasion. Manipulation of ECM density was associated with an altered migration pattern from glandular buds (cellular aggregates) to epithelial cell sheets. Laminin appeared to be a critical component in regulating endothelial cell morphology and vascular network formation. Interestingly, the disruption of vascular networks by cancer cells was driven by changes in expression of several anti-angiogenic genes. Cancer cells cultured in our biomimetic tumouroids exhibited intratumoural heterogeneity that was associated with increased tumour invasion into the stroma. These findings demonstrate that our 3D *in vitro* tumour model exhibits biomimetic attributes that may permit their use in studying microenvironment clues of tumour progression and angiogenesis.

Despite the significant advancements in early diagnostic and therapeutic regimens, the metastatic progression of tumours is the leading cause of mortality in colorectal cancer patients^1^. Tumour progression is mediated by microenvironmental conditions that include oxygen gradients between tumour cells in spatially distinct regions, cell-cell and cell-extracellular matrix (ECM) interactions^2^. Understanding the more complex mechanics of tumour cell migration within conventional 2D *in vitro* models has proved challenging and as a result, there has recently been an increase in tissue engineered solutions to address this problem^3^,^4^. One avenue, not often explored within 3D *in vitro* tumour models, is the effect of the tumour stroma on cancer growth and invasion. ECM density and composition are factors that are often overlooked in cancer research but have increasingly been implicated as significant factors involved in cancer progression^5^.

Natural scaffolds are composed of ECM components that make up an interlocking mesh of fibrous proteins and glycosaminoglycans (GAGs) including collagens, fibrin and hyaluronic acid^6^,^7^. They provide tissues and cells with mechanical stability and enable cell-matrix interactions to regulate normal tissue function. Natural scaffolds are also biologically active and promote excellent cell adhesion, growth and migration^8^. When used for *in vitro* 3D cell culture, these scaffolds exist as cross-linked networks of ECM proteins known as hydrogels. Although one of their main disadvantages is their high water content (upwards of 99%), they are still extremely useful for mechanistic investigations as they are entirely malleable by cell behaviour and subject to cell mediated ECM degradation. Increasing the matrix density of these scaffolds can help recreate normal or pathological tissue function.

We engineered tumouroids using colorectal cancer cells (HT29 or HCT116) and cultured them within collagen type I hydrogels. To increase the matrix density and mimic the dense nature of *in situ* tumours, the interstitial fluid within collagen hydrogels was removed using plastic compression (PC)^9^. Tumouroids are spatially accurate and are based on a dense central artificial cancer mass (ACM) that contains the cancer cells, nested within a collagen hydrogel that represents the tumour stroma (Fig. 1a). The stromal compartment was populated with the basement membrane protein and attachment factor laminin, and stromal cells such as fibroblasts and endothelial cells (ECs). The effect of matrix density and composition on cancer invasion was investigated. The development of ‘healthy’ and ‘tumourigenic’ vascular networks in the stroma was also explored due to the presence of the endothelium adjacent to a tumour in the *in vivo* scenario. The current work presented here focuses on developing more biomimetic tumour models, which incorporate important aspects of the tumour microenvironment that hopefully will shed light on novel mechanisms involved in cancer progression.

## Results

### Extracellular matrix density and composition of the stroma regulates cancer cell invasion

**Figure 1:**
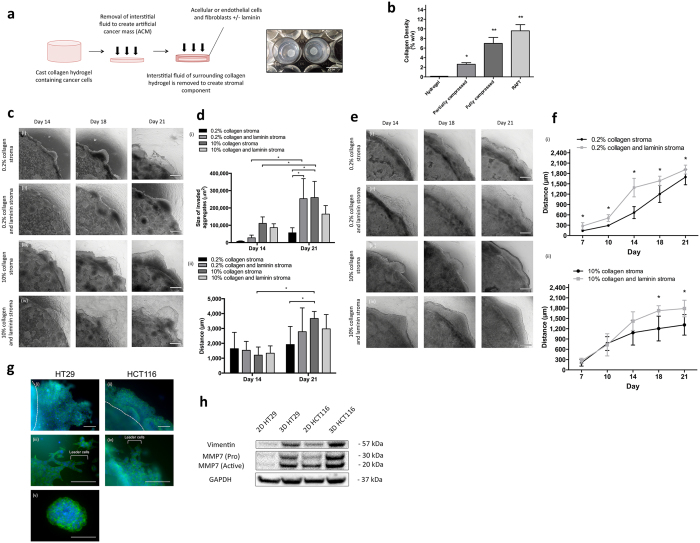
Extracellular matrix density and composition of the stroma regulates cancer cell invasion. (**a**) A schematic diagram illustrating how biomimetic tumouroids are created and the gross appearance of the ACM containing HT29 cells nested into the stromal surround, which is either acellular or contains HDFs and HUVECs. (**b**) The matrix densities of collagen gels (n = 4) as a % w/v collagen concentration under different compressions, uncompressed, 175 g weight for 1 minute, 175 for 10 minutes and hydrophilic RAFT absorbers for 15 minutes. (**c**) HT29 cells cultured in a high-density collagen matrix (i) invade into a low-density collagen stroma and a (ii) low-density collagen and laminin stroma, both as cellular aggregates. (iii) HT29 cell invasion switches to an epithelial cell sheet in a high-density collagen matrix stromal surround and (iv) invasion is enhanced when laminin is added to the stroma. (**d**) Quantification of the (i) size of invading cellular aggregates and (ii) the distance that cellular aggregates invaded into each respective stromal surround. (**e**) HCT116 cells cultured in a 10% collagen matrix (i) invade into a 0.2% collagen stroma as cell sheets and a (ii) 0.2% collagen and laminin hydrogel stromal surround. (iii) HCT116 cells invade into a 10% collagen only stromal surround and (iv) enhanced invasion when laminin is present. (**f**) Quantification of the total distance invaded by HCT116 cells (i) into a 0.2% collagen stromal surround (ii) and a 10% collagen matrix stromal surround. (**g**) The different morphologies of HT29 and HCT116 cells invading into an acellular stroma. (**h**) The expression of pro-invasive and EMT markers MMP7 and vimentin in 2D monolayers compared to 3D tumouroids cultured for 10 days. Data is presented as mean ± SD (n = 6). *p < 0.05. Scale bars – 500 μm, (magnified views) 100 μm.

The matrix density of collagen gels was evaluated by freeze-drying acellular collagen gels under a variety of PC methods. Partially and fully compressed gels were prepared as described previously and were compressed with a 175 g weight for a total of 1 minute and 10 minutes^10^. Partial compression of the collagen gels revealed a 13-fold increase (2.63 ± 0.32%) in collagen density in comparison to standard uncompressed collagen gels (0.2%)(p < 0.05)(Fig. 1b). Full compression using this method revealed a 35-fold increase in collagen density (6.98 ± 1.24%) in comparison to collagen hydrogels. RAFT absorbers produced a matrix with a 48-fold increase in collagen density (9.59 ± 1.28%) (p < 0.0001) in comparison to uncompressed collagen gels. Uncompressed collagen hydrogels are initially set at ~2.5 mg/ml of collagen, following compression the protein concentration also increases ~48 fold to 120 mg/ml.

We engineered ACMs of 10% (w/v) collagen (Fig. 1a and b) containing either HT29 or HCT116 cells embedded in either 0.2% or 10% matrix density stroma. Cells formed cellular aggregates within the ACM mimicking avascular micrometastases and invaded the stroma in cell specific patterns (Fig. 1c). HT29 cells invaded as cellular aggregates in 10% matrix tumouroids (Fig. 1c). Cellular aggregates detached from the ACM as they migrated into the 0.2% collagen stromal surround (Fig. 1ci and ii) forming a network of budding glandular structures resembling tumour budding observed at the invasive front of *in situ* tumours^11^. The addition of the attachment factor and basement membrane (BM) constituent laminin increased the size of the invading aggregates at day 21 between collagen only (56,615.9 μm^2^ ± 28,792.6 μm^2^) and collagen and laminin stromal cultures (253,564.6 μm^2^ ± 115,601.8 μm^2^) (Fig. 1di) (p < 0.05) as measured by ImageJ analysis (Supplementary Figure 1). This is likely due to the earlier onset of invasion whereby cells migrate to regions of higher nutrients and O_2_. When the matrix density of the surrounding stroma was increased from 0.2% to 10% collagen, HT29 cells demonstrated additional migratory patterns (Fig. 1ciii and iv). While cellular aggregates were still distinguishable within the stroma, contiguous cell sheets also invaded from the ACM into the stroma. Invading cellular aggregates displayed a trend of migrating further in the dense stromal surround in comparison to hydrogels (Fig. 1dii).

HCT116 cells, which are more metastatic than HT29 cells^12^, invaded exclusively as epithelial cell sheets (Fig. 1e) under all stromal conditions. The presence of invading cell sheets mimics epithelioid neoplastic cell sheet invasion at the tumour-stroma boundary in medullary carcinomas, which have an occurrence rate of 5–8 cases for every 10,000 colorectal cancers diagnosed^13^. The addition of laminin to the 0.2% collagen stromal cultures increased the overall migration distance of the epithelial cell sheets between day 7 and day 21 in comparison to collagen only stromal surrounds (Fig. 1f). Interestingly, when the distances of cell sheet invasion were quantified in different stromal surrounds, highly different patterns of the rate of invasion were observed. In the 0.2% collagen stroma, invasion of the cell sheets appeared slow at first, however between day 10 and day 21, the rate of invasion increased in a linear fashion. On the contrary, the invasion pattern of HCT116 cells migrating into a 10% collagen stromal surround was earlier than the 0.2% collagen stroma. Conversely, at day 14, HCT116 cell sheet invasion appears to slow down significantly until day 21 where the presence of laminin appears to be the major driving factor in enhancing the total distance migrated.

Invading HT29 cells displayed three different morphologies whereas HCT116 cells displayed only two (Fig. 1g). Although the location and morphology of the invading cells appeared random and unsystematic, there were distinguishable features to each pattern. In some instances, HT29 cells protruded outwards from the ACM into the stromal surround and formed coordinated one-directional organized migration of the invading cell sheet (Fig. 1gi). On the other hand, invading HCT116 cell sheets had no structured organization and the migration appeared random in all directions (Fig. 1gii). Moreover, the morphology of the cells at the tip of the invading sheets differed greatly from those comprising the bulk of the cell sheet. These ‘leader cells’ were particularly elongated for both HT29 and HCT116 tumouroids, and appeared morphologically distinct from the cells within the ACM (Fig. 1giii and iv). Additionally, HT29 cells also formed cellular aggregates, which completely detached from the ACM and invaded into the stroma (Fig. 1gv). This mode of migration was entirely absent from HCT116 tumouroids.

In order to validate phenotypic changes associated with cancer cell invasion, we examined the presence of EMT and MMP expression involved in ECM degradation. Western blotting was used to detect the expression patterns of MMP7 and the EMT marker vimentin between cells cultured in 2D and in tumouroids (Fig. 1h). Vimentin expression was highly upregulated in both HT29 and HCT116 tumouroids in comparison to their monolayer counterparts. The expression level of active MMP7 in HT29 cells was four times higher in tumouroids than in 2D. On the other hand, HCT116 tumouroids and 2D monolayers did not illustrate a dramatic difference in active MMP7 expression. MMP7 degradation of the β3 chain in laminin-5 enhances the migration of colorectal cancer cells^14^, therefore it is possible the upregulation of MMP7 coupled with the presence of laminin in the stroma is responsible for the enhanced migratory profile of both HT29 and HCT116 tumouroids. MMP7 overexpression has been linked to an increased incidence of metastasis and advanced disease in colorectal cancer^15^,^16^. Although MMPs are typically expressed by stromal cells, MMP7 was chosen due to its established expression exclusively by carcinoma cells^17^.

### Disrupted vascular networks in 3D biomimetic tumouroids

**Figure 2:**
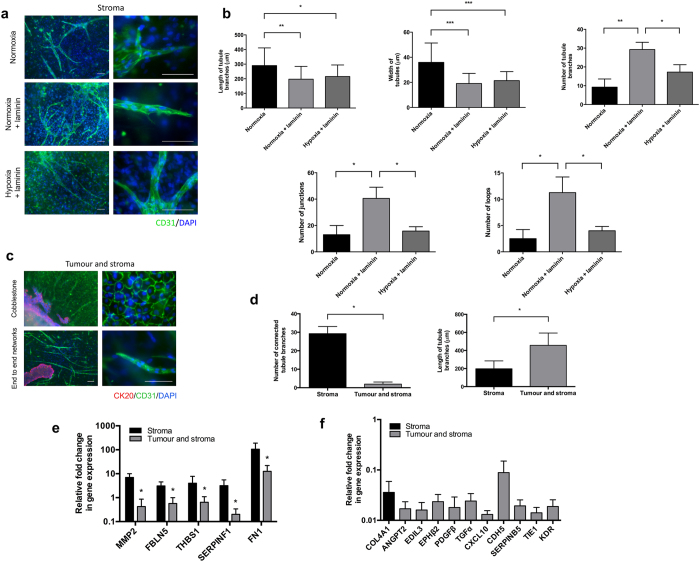
Characterization of *in vitro* healthy and tumourigenic vasculature. (**a**) HDFs and HUVECs (stromal only cultures) were cultured for 21 days under normal atmospheric oxygen (normoxia ~21% O_2_) in 10% collagen gels with and without laminin and in physiological hypoxia (~5% O_2_) with laminin only. EC morphology was assessed using immunofluorescence of CD31 (green) and DAPI (blue). (**b**) ImageJ was used to quantify the length, width, and number of branches, loops and junctions of vascular networks in the stromal only cultures. Normoxia cultures without laminin formed longer and wider networks than in the presence of laminin, however this may have been due to the presence of the EC cobblestones, which led to a significantly lower level of connectivity among the branches. Stromal cultures containing laminin had a significantly higher number of branches, loops and junctions in comparison to normoxia without laminin and hypoxia with laminin. (**c**) In the presence of cancer cells (biomimetic tumouroids), ECs formed two distinct morphologies, cobblestones and end-to-end vascular networks in the stroma as confirmed by immunofluorescence of CD31 (green). HT29 cells were stained for CK20 (red) while the unstained nuclei in the stroma are HDFs. (**d**) ImageJ was used to analyze the differences in tubule length and branch connectivity in the presence and absence of HT29 cancer cells and showed vascular network integrity was maintained in the absence of HT29 cancer cells. (**e**) Quantitative RT-PCR analysis of angiogenic genes downregulated in biomimetic tumouroids in comparison to the stromal only cultures and (**f**) the expression profiles of angiogenic genes expressed solely in either the stroma or in biomimetic tumouroids. Data is presented mean ± SD (n = 6). *p < 0.05, **p < 0.01, ***p < 0.0001. Scale bar – 100 μm.

To evaluate the vasculogenic potential of the ECs, we co-cultured them in either the presence of HDFs only (stromal cultures alone) or HDFs and HT29 cancer cells (biomimetic tumouroids). In the absence of laminin, ECs formed end-to-end vascular networks in the stromal only cultures (Fig. 2a). These vascular networks were longer and wider, but considerably less interconnected than the stromal only cultures in the presence of laminin (Fig. 2b). Vascular networks in the stromal cultures without laminin had a length and width of 290.3 μm ± 120.7 μm and 35.9 μm ± 15.4 μm respectively whereas the presence of laminin led to the formation of vascular networks with a length and width of 197.5 μm ± 87.5 μm and 19.1 μm ± 8.1 μm correspondingly (p < 0.05) (Fig. 2b). Stromal cultures in physiological hypoxia with laminin had a similar length and width to the normoxia stromal cultures (215.2 μm ± 79 μm and 21.4 μm ± 7.2 μm) (Fig. 2b). Interestingly, the presence of laminin in the normoxia stromal cultures appeared to produce much more significantly interconnected vascular networks as signified by the large number of branches, loops and junctions (p < 0.05) (Fig. 2b).

The presence of HT29 cancer cells led ECs to aggregate in a cobblestone pattern within the stroma of the normoxia cultures (Fig. 2c). In addition to the cobblestone morphology, HUVECs formed significantly longer vascular branches in comparison to the stromal cultures alone (456.8 μm ± 136 μm compared to 197.4 μm ± 87.5 μm) (p < 0.0001) (Fig. 2d). While these tubules were over twice as long, they were significantly less interconnected (29.3 ± 3.8 compared to 1.9 ± 1.1 branches) (p < 0.0001). Cobblestone cells formed in large sheets on the apical side of the collagen gel forming a monolayer and strongly expressing membrane bound CD31 while they also formed end-to-end networks. These two morphologies mimic developmental mechanisms for tubulogenesis such as wrapping and cell hollowing^18^. This EC morphological heterogeneity is likely due to the chronic exposure to pro-angiogenic factors that we have shown before^10^, which leads to the deregulation of the neovascularization process.

We then compared the expression profile of a variety of angiogenic genes between the stroma only cultures and the biomimetic tumouroids to elucidate possible differences in gene expression responsible for the formation/disruption of the formation of vascular networks. Of the 92 genes tested, 5 were differentially expressed in both the stromal cultures and the biomimetic tumouroids. Interestingly, *MMP2, FBLN5, THBS1, SERPINF1 and FN1* were significantly downregulated in biomimetic tumouroids in comparison to the stromal cultures (Fig. 2e). Three of these genes, *FBLN5, THBS1* and *SERPINF1* are important anti-angiogenic factors and are known to inhibit tumour growth, increase vascular permeability and decrease vascular sprouting^19^,^20^,^21^. The most interesting of these, *SERPINF1*, which codes for the Pigment epithelium-derived factor (PEDF), is the most potent inhibitor of angiogenesis discovered to date and is twice as potent as angiostatin and seven times more potent than endostatin^22^. Furthermore, 10 genes were expressed exclusively in biomimetic tumouroids (Fig. 2f), including *EDIL3* which is involved in the formation of complex vascular like structures. Its expression correlates with vascular remodelling and poor vascular wall integrity observed in the chick chorioallantoic membrane and functions via binding to the αVβ3 integrin receptor^23^. The same study showed that *EDIL3* expression entirely prevents ECs from forming vascular like structures *in vitro*. Interestingly, the formation of vascular networks was not entirely hindered in our tumouroids and we have previously shown a novel mechanism of vascular network formation dependent on α6 integrin expression. α6 integrin is critical for cell-laminin attachment and regulates *VEGFR2* levels to promote end to end vascular network formation of ECs in collagen gels^24^. Furthermore, the qPCR analysis also revealed the overexpression of two important negative regulators of angiogenesis. *ANGPT2* and *TIE1* contribute to normal vascular disruption via inhibition of ANG1 signalling through the formation of a *TIE1/TIE2* heterotypic complex. *ANGPT2* and *TIE1* are specifically upregulated in tumour vasculature, highlighting the similarity in biomarker expression between these 3D biomimetic tumouroids and *in vivo* tumours^25^,^26^.

### Tumour heterogeneity and vascular network interactions in 3D biomimetic tumouroids

**Figure 3:**
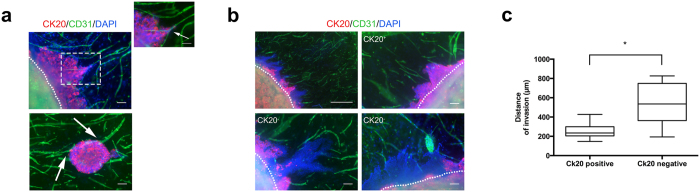
Vascular network interaction with cancer cells and intratumoural heterogeneity of biomimetic tumouroids. (**a**) Biomimetic tumouroids were cultured for up to 21 days and stained for CK20 (red), CD31 (green) and DAPI (blue). Vascular networks appeared to migrate towards the aggressive invading epithelial cell sheet tip where they diverged (white arrow, top right panel). Singular tubule branches also migrated towards the invaded aggregates within the stroma (white arrow, bottom panel). The white dotted lines denote the boundary of the ACM and the stromal surround. (**b**) CK20 expression was lost in invading HT29 cells and (**c**) the loss of CK20 correlated with the overall distance migrated of the invading epithelial cell sheet. Data is presented as mean ± SD (n = 6). *p = 0.0089. Scale bar (top) – 1000 μm, (bottom) – 100 μm.

Tumour induced angiogenesis is driven by a series of cell-cell and cell-ECM interactions between cancer cells and ECs. Therefore, the following experiments attempted to visualize some of these cell-cell interactions. HT29 cells and ECs were stained for CK20 and CD31 respectively. Figure 3a illustrates cell-cell specific interactions between the vascular networks and the cancer cells. While the vascular networks in the biomimetic tumouroids did not contain any junctions or highly branched networks, they appeared to fork into separate or diverging tubules that appeared to grow directly into the invading cell sheet (Fig. 3a, inset).

As solid tumours grow in a 3D spatial configuration, the cells within a tumour are exposed to varying levels of oxygen and nutrients. This leads to physical and chemical stresses that regulate differential gene expression of cells in different regions within the same tumour. This intratumoural heterogeneity is more analogous to the *in vivo* situation where diffusion limitations result in regions of hypoxic and proliferating cells^27^. We investigated the expression of CK20 as a colorectal cancer marker, which is involved in the identification of normal intestinal epithelium and adenocarcinomas and is used routinely in the clinic^28^. While evaluating the interactions between cancer cells and the surrounding vascular networks, we observed the loss of CK20 in some of the invading cell sheets within the same tumouroid. However, this was strictly limited to the invading epithelial sheets that had migrated the furthest distance from the ACM (Fig. 3c) indicating that loss of CK20 was associated with a significant increase in the overall distance of cancer cell invasion. This increase in invasion was taken as an indicator of tumour aggressiveness. CK20 negative sheets invaded an average distance of 516.6 μm ± 224.3 μm whereas HT29 cells that retained CK20 expression invaded 254.9 μm ± 84.3 μm (p = 0.0089). HT29 aggregates within the ACM still expressed strong levels of CK20, highlighting the heterogeneity of protein expression within the same tumouroid (Fig. 3b, top left).

## Discussion

We have described the development of a 3D *in vitro* tumour model with controllable parameters including ECM density and composition of the tumour and stroma. While cell migration is a highly coordinated process, we have shown that in the *in vitro* setting, it is highly dependent on ECM composition over ECM density. Laminins are major regulators of cell adhesion, migration and proliferation^24^,^29^. They regulate these cellular processes via specific integrin binding sites that switch on mechanotransduction pathways to promote cancer cell invasion. Specifically, the laminin-5/laminin-332 γ2 chain has been implicated as an adhesion substrate for epithelial cells and is expressed in a wide variety of invasive carcinomas^30^. Furthermore, the overexpression of the EMT marker vimentin and MMP7 suggests that cancer cell invasion in our tumouroids is an active process dependent on the proteolysis of the surrounding ECM. To date, most studies have only demonstrated immunohistochemically the co-localization or overexpression of specific laminin sub-units at the invasive front of tumours^31^. While there is little mechanistic data directly linking the presence of laminins and increased invasion, our findings are in agreement with recent studies showing MMP7 degradation of the β3 chain in laminin-5/laminin-332 enhanced the migration of colorectal adenocarcinoma^14^. It is likely the upregulation of MMP7 coupled with the addition of laminin in the stroma is responsible for the enhanced invasion of both HT29 and HCT116 cells in our tumouroids.

Surprisingly, it appears the invasion process of cancer cells is driven by several mechanisms simultaneously as seen by the presence of glandular structures, polarized collective migration and cell sheets directed by elongated cells. The presence of glandular buds mimics what is observed in patients with CRC. Zlobec *et al*. developed an assay using full histopathological data from preoperative biopsies taken from patients with CRC to identify whether tumour budding could be used as a predictive method for identifying lymph node and distant metastasis^11^. Tumour cells isolated from patient biopsies could be cultured in our tumouroid model and graded alongisde standard histopathological analyses to predict tumour invasiveness. Furthermore, we have also identified different mechanisms of migration (glandular buds vs cell sheets) between different colorectal cancer cell lines, independent of ECM density or composition, driven in part by a transformation towards a mesenchymal lineage. Interestingly, elongated cells with a mesenchymal morphology at the edge of the invading cell sheet formed cellular protrusions. These leader cells often guide the following cells that compose the main body of the cell sheet. The collective migration of these cells did not appear to be driven in a single direction, with the presence of many leader cells migrating in random directions and appearing non-polarized. Several recent studies have highlighted the importance of these leader cells and their presence on the invasive front, and it is thought that they are phenotypically different from follower cells^14^,^32^. A recent study by Cheung and colleagues demonstrated in a 3D organoid model of breast cancer that collective invasion was led by genotypically distinct cells that were defined by their expression of the basal epithelial genes K14 and p63^33^. They showed that knockdown of either K14 or p63 was sufficient enough to block collective invasion. Although, the behaviour of leader cells is not well characterized in cancer, it is likely to mimic TGFβ stimulated collective migration which occurs during the wound healing process^32^.

*In situ*, the metastatic cascade is initiated through a series of interactions between tumour cells and the nearby endothelium. While there is no functioning endothelium within our tumouroids, the same cell-cell interactions that take place within the *in vivo* microenvironment between cancer cells and ECs are present, evident by the formation of ‘healthy’ and tumourigenic vascular networks. We have shown in this study that vasculogenic and angiogenic processes are not entirely mediated by oxygen gradients and the ECM protein laminin is critical in regulating EC behaviour. This emphasizes the importance of matrix composition in regulating vasculogenesis and angiogenesis. Stamati and colleagues demonstrated end-to-end networks in a model of vasculogenesis in collagen hydrogels using HUVECs and human bone marrow derived stem cells (HBMSCs)^24^. The authors identified an important link between increased VEGFR2 production on ECs, regulated by HBMSCs, which lead to key aggregation patterns in collagen gels in the presence of basement membrane components. It is likely that while no HBMSCs were used in this study, the HDFs provided the ECs with a cocktail of angiogenic growth factors to generate end-to-end networks. This opens up a new avenue of therapies which may be able to inhibit EC-laminin attachment by blocking specific α6 integrin binding sites.

Intratumoural heterogeneity plays a important role in the clinical outcome of response to therapy^34^. This heterogeneity is complex and poorly understood in the clinic. Therapeutic resistance is mainly caused by the presence of many drug-resistant variants of cancer cells within the primary tumour and it has been shown that these drug resistant variants are those which repopulate a tumour following neoadjuvent therapy^35^. In our HT29 tumouroids, the loss of CK20 expression in the invading epithelial cell sheets strongly correlated with the overall distance migrated of the invading sheet indicating that loss of CK20 was associated with tumour aggressiveness. Whether the loss of CK20 is a predictive marker for this increased invasiveness or just a by-product of this genetic variant is of great importance in understanding the clinical behaviour of colorectal tumours. The loss of CK20 in the clinical setting is drastically correlates with microsatellite instability, higher tumour grade, poor differentiation and overall poor survival^36^,^37^. We believe that this is the only study to recapitulate the loss of CK20 in CRC cell lines and directly correlate it with increased invasion in a 3D *in vitro* tumour model.

Although our tumouroids lack the full complexity of animal models, this model is cost and time effective, allows for easy manipulation of cell and matrix densities and exhibits characteristics of the native *in vivo* tumour microenvironment. Biomimetic 3D tumour models present alternative tools to investigate the effect of multiple cell populations and the role of the ECM in cancer progression.

## Materials and Methods

### Cell Culture

The HT29 human colorectal adenocarcinoma cell line and the HCT116 human colorectal carcinoma cell lines were obtained from the European Collection of Cell Cultures (Sigma Aldrich, Dorset, UK). Human umbilical vein endothelial cells (HUVECs) were purchased from Promocell (Heidelberg, Germany) and Human dermal fibroblasts (HDFs) from Invitrogen (Paisley, UK). HT29 and HCT116 cells were cultured in Dulbecco’s Modified Eagle Medium (DMEM) supplemented with 1 g/L glucose, 10% FBS, 100 units/ml penicillin and 100 μg/ml streptomycin (all from Invitrogen, Paisley, UK). HUVECs were cultured in complete endothelial growth medium (EGM) (Promocell, Heidelberg, Germany) supplemented with 10% FBS and 100 units/ml penicillin and 100 μg/ml streptomycin. HDFs were cultured in high glucose (5 g/L) DMEM supplemented with 10% FBS and 100 units/ml penicillin and 100 μg/ml streptomycin. All cell types were routinely maintained as 2D monolayers at 37 °C in standard cell culture conditions (5% CO_2_/air and 95% humidity).

### Fabrication of 3D biomimetic tumouroids

The ACMs were prepared using the RAFT™ 3D cell culture system (Lonza, Slough, UK) as described previously^38^. Briefly, 2.8 ml of 10X minimum essential medium (MEM) was added to 22.4 ml rat-tail collagen type I (2.05 mg/ml in 0.6% acetic acid). This was neutralized with 1.6 ml of the Neutralizing Solution before 1.2 ml of the cancer cell suspension (HT29 or HCT116) at a concentration of 4.96 × 10^6^ cells/ml, was added and mixed thoroughly. Gels were cast in 96 well plates (240 μl) and placed on a plate heater (Sartorius, Royston, UK) set at 37 °C for 15 minutes. Each ACM contained 50,000 HT29 or HCT116 cells per well (208,000 cells/ml). Biocompatible hydrophilic RAFT absorbers were placed on the gels for 15 minutes to remove the interstitial fluid from the collagen gels. The absorbers were removed, 100 μl of culture medium was added to the wells and the plate was returned to the incubator (37 °C, 5% CO_2_/air and 95% humidity).

To incorporate laminin into the acellular stroma, 50 μg/ml of laminin (BD Biosciences) was added to the collagen-MEM mixture prior to neutralization. This concentration was based on published literature^24^. Briefly, 500 μl of neutralized acellular collagen ± laminin was cast in 24 well plates and allowed to gel for 5 minutes on a plate heater at 37 °C. Each ACM was then removed manually from their 96-well plate then placed on the gel before 500 μl of acellular neutralized collagen ± laminin was added on top to fully immerse the ACM. This was allowed to gel for 10 minutes at 37 °C. These cultures were used as 0.2% cultures. For 10% matrix cultures the interstitial fluid was removed using hydrophilic RAFT absorbers and the tumouroids were immersed in DMEM.

To engineer the cellular stroma, HUVECs and HDFs were added to the neturalized collagen-laminin solution at a concentration of 50,000 and 25,000 cells respectively per single tumouroid resulting in ratio of 8:2:1 for HT29, HUVEC and HDF cells (Fig. 1a).

Stromal only cultures were fabricated with the same ratio of 2:1 of HUVECs to HDFs in the absence of ACMs. Finally, 1 ml of DMEM and EGM mix (1:1) was added to each well and returned to the incubator. Experiments carried out in physiological hypoxia were carried out in a separate incubator set at 37 **°**C, 5% CO_2_, 5% O_2_ and 90% N_2_. Physiological hypoxia is also known as ‘*in situ* normoxia’ and is representative of the normal O_2_ pressure present within tissues.

### Immunofluorescence

Tumouroids were fixed using 10% formalin for 30 minutes and washed with PBS. After permeabilising with 0.2% TX- 100 for 30 minutes, the gels were washed thoroughly with PBS (3 × 5 minutes) and blocked with 1% BSA for 30 minutes. After washing with PBS, gels were incubated with either a CK20 rabbit primary antibody (D9Z1Z from New England Biolabs, Herts, UK) or an anti-CD31 mouse primary antibody (JC70/A from Abcam, Cambridge, UK) overnight at 4 °C. After washing thoroughly, gels were incubated with an Alexa Fluor 488 goat anti mouse IgG secondary antibody (Invitrogen, Paisley, UK) and DyLight 594 goat anti-rabbit IgG secondary antibody (Vector labs, Peterborough, UK) for 2.5 hours at room temperature. The actin filaments were stained with Rhodamine Phalloidin conjugated to an Alexa Fluor^®^ 488 fluorescent antibody (Invitrogen) for 1 hour at room temperature. The gels were then washed with PBS thoroughly and 2 drops of NucBlu (Invitrogen, Paisley, UK) was added to the PBS in each sample. Tumouroids were imaged using an inverted EVOS^®^ FL imaging microscope (Invitrogen).

### Collagen gel density measurement

The density of collagen gels was assessed using the freeze-drying method. Acellular collagen gels were prepared and compressed under various weights. Collagen gels (uncompressed), partially compressed gels (compressed with 175 g weight for 1 minute), fully compressed gels (compressed with 175 g weight for 10 minutes) and RAFT gels (removal of interstitial fluid using hydrophilic RAFT absorbers for 15 minutes). Once the gels were prepared, DMEM was added to each sample for 24 hours to accommodate a bounce back effect from the collagen gels (reabsorption of fluid). The gels were then washed with PBS followed by a thorough wash in distilled water in until all the salts within the gel were removed. This was estimated by a colour change of the collagen gel from pink to colourless. The gel weight was then measured using a balance to calculate the wet weight of the gel. Each gel was then frozen at −20 °C for 1 hour before being freeze-dried overnight and the weight of the dry gels was measured again. The difference between the wet and dry weights was then used to calculate the overall collagen content within the gels once the water had been removed.

### Western blotting

Cells were extracted from tumouroids using 500 units/ml collagenase (Sigma Aldrich) for 1 hour at 37 °C. Cells in 2D and 3D were lysed in RIPA buffer containing protease inhibitors (Sigma) and quantified using the Pierce™ Modified Lowry Protein Assay Kit (Thermo Scientific) according to the manufacturers protocol. Equal amounts of protein (35 μg) were loaded in gels (BioRad), resolved by SDS-PAGE and transferred to PVDF membranes. Membranes were blocked for 30 minutes in 2.5% BSA and 0.05% Tween 20 in PBS. Antigens were detected using mouse monoclonal antibodies against MMP-7 and vimentin (both at 1:1,000, Santa-Cruz Biotechnology, Santa Cruz, CA) and incubated with a goat anti-mouse IgG-HRP (Santa Cruz) secondary antibody at room temperature for 30 minutes. Blots were developed using the Clarity™ Western ECL Substrate (Bio-Rad, Hertfordshire) and visualized using the ChemiDoc™ XRS+ System (Bio-Rad).

### Real-time angiogenesis PCR array

Total RNA was isolated from stromal only cultures (HDFs and HUVECs only) and biomimetic tumouroids (ACM and stroma) using TRI Reagent^®^ (Sigma) according to the manufacturers instructions. Total RNA was quantified using a nanodrop spectrophotometer measuring at 260/280 nm. cDNA was synthesized using the High Capacity cDNA Reverse Transcription kit (Applied Biosystems) according to the manufacturers instructions. Gene expression analysis was conducted using the TaqMan^®^ Human Angiogenesis PCR array (Applied Biosystems). This assay contains 92 genes related to angiogenesis and lymphangiogenesis (see www.thermofisher.com for a full list of the genes). Gene expression levels were measured by RT-PCR using the CFX96 Touch system (Bio-Rad) following the array manufacturers instructions. Relative gene expression was calculated using the 2-Δ*C*_T_ method. Changes in gene expression are presented as fold increase/decrease normalized to the average CT value for the endogenous reference genes Beta-glucuronidase (GUSB), 18S ribosomal RNA (18S) and Glyceraldehyde 3-phosphate dehydrogenase (GAPDH).

### Statistical analysis

The data presented throughout are displayed as the mean ± standard deviation (SD) and was calculated using GraphPad Prism 6 software (GraphPad, San Diego, CA). Statistical analysis was carried out on original data values where the data was converted to percentages or fold increases/decreases. Data points (n numbers) are displayed for each respective experiment in the figure legend. Data comparisons for two sets of data were analyzed for statistical significance by Students t-test analysis. Vascular network analysis (Supplementary Figure 1c) among different groups was carried out using one-way analysis of variance (ANOVA) followed by post hoc analysis using Tukeys multiple comparisons test. Gene expression analysis was conducted using two-way ANOVA followed by post hoc analysis using Sidak’s multiple comparisons test. Significance was taken at or below p < 0.05.

## Additional Information

**How to cite this article:** Magdeldin, T. *et al*. Engineering a vascularised 3D *in vitro* model of cancer progression. *Sci. Rep.* **7**, 44045; doi: 10.1038/srep44045 (2017).

**Publisher's note:** Springer Nature remains neutral with regard to jurisdictional claims in published maps and institutional affiliations.

## Supplementary Material

Supplementary Information
