# Supplementary material for: Engineering a vascularised 3D in vitro model of cancer progression
Source: Sci Rep. 2017 Mar 9;7:44045. doi: 10.1038/srep44045 (PMC5343474; doi:10.1038/srep44045)
Supplement: Supplementary Information [file srep44045-s1.pdf]

Engineering a vascularised 3D *in vitro* model of cancer progression

Tarig Magdeldin[1, 2], Víctor López-Dávila[2], Judith Pape[1], Grant W. W. Cameron[3], Mark Emberton[2], Marilena Loizidou[2], Umber Cheema[1*]

[1] UCL Institute of Orthopaedics and Musculoskeletal Sciences, UCL Division of Surgery and Interventional Science, Stanmore Campus, HA7 4LP, U.K.
[2] UCL Division of Surgery and Interventional Science, Royal Free Campus, London, NW3 2QG, U.K.
[3] Sartorius Stedim Biotech, Royston, Herts, SG8 5WY, U.K.

*** Corresponding author:** Dr Umber Cheema, UCL Institute of Orthopaedics and Musculoskeletal Sciences, UCL Division of Surgery and Interventional Science, Stanmore Campus, HA7 4LP, U.K. Email: u.cheema@ucl.ac.uk

Supplementary Figure 1: Image analysis of cancer cell aggregates, cancer cell sheets and vascular networks.

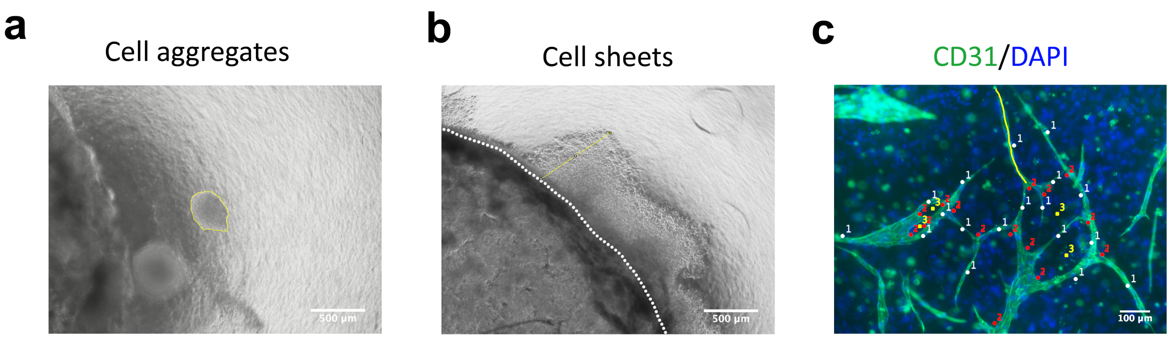

(a) The total surface area (µm^2) of cell aggregates was measured using the ImageJ software version 1.47 (National Institute of Health, USA). Cancer invasion was defined as cell aggregates detaching and invading the stromal surround or cell sheets growing outwards from the ACM. The freehand line tool was used to trace around the HT29 cellular aggregates and the surface area was calculated using a pre-set scale set in ImageJ. (b) HCT116 epithelial cell sheet invasion was measured using the straight-line tool. The furthest distance the cell sheet had migrated from the ACM was taken as a single measurement. (c) For vascular networks the length, width, number of branches, number of junctions and loops were quantified using the cell counter plugin for ImageJ. The length of each branch was measured and traced over using the freehand line tool (yellow line). The numbers in the images below signify how the number of branches (number 1), junctions (number 2) and loops (number 3) were counted. The width of each vascular branch was measured using the straight-line tool and drawn across the width of each vascular branch within the image. Six images were analyzed per condition and all tubules, junctions and loops were quantified. All analyses were carried out manually. Scale bars – 500µm, 100µm (right).
